# Catalytic activity of nanostructured Au: Scale effects versus bimetallic/bifunctional effects in low-temperature CO oxidation on nanoporous Au

**DOI:** 10.3762/bjnano.4.13

**Published:** 2013-02-19

**Authors:** Lu-Cun Wang, Yi Zhong, Haijun Jin, Daniel Widmann, Jörg Weissmüller, R Jürgen Behm

**Affiliations:** 1 Institute of Surface Chemistry and Catalysis, Ulm University, D-89069 Ulm, Germany; 2 Institut für Werkstoffforschung, Helmholtz-Zentrum Geesthacht, D-21502 Geesthacht, Germany; 3 Institute of Metal Research, Chinese Academy of Sciences, 110016, Shenyang, P.R. China; 4 Institut für Werkstoffphysik und Werkstofftechnologie, TU Hamburg-Harburg, D-21073 Hamburg, Germany

**Keywords:** AuAg alloy, AuCu alloy, CO oxidation, dynamic studies, kinetics, nanoporous Au (NPG) catalyst, oxygen storage capacity (OSC), temporal analysis of products (TAP)

## Abstract

The catalytic properties of nanostructured Au and their physical origin were investigated by using the low-temperature CO oxidation as a test reaction. In order to distinguish between structural effects (structure–activity correlations) and bimetallic/bifunctional effects, unsupported nanoporous gold (NPG) samples prepared from different Au alloys (AuAg, AuCu) by selective leaching of a less noble metal (Ag, Cu) were employed, whose structure (surface area, ligament size) as well as their residual amount of the second metal were systematically varied by applying different potentials for dealloying. The structural and chemical properties before and after 1000 min reaction were characterized by scanning electron microscopy (SEM), X-ray diffraction (XRD) and X-ray photoelectron spectroscopy (XPS). The catalytic behavior was evaluated by kinetic measurements in a conventional microreactor and by dynamic measurements in a temporal analysis of products (TAP) reactor. The data reveal a clear influence of the surface contents of residual Ag and Cu species on both O_2_ activation and catalytic activity, while correlations between activity and structural parameters such as surface area or ligament/crystallite size are less evident. Consequences for the mechanistic understanding and the role of the nanostructure in these NPG catalysts are discussed.

## Introduction

Porous metallic materials with well-controlled morphologies and surface properties have attracted considerable attention in both fundamental research and technological applications owing to their unique physical and chemical properties, for applications, e.g., in optics, catalysis or as sensors [1–2]. Among these materials, monolithic gold with nanoporous structures as well as high surface areas is of particular interest for catalysis research in view of its self-supporting nature [3–7]. This kind of material is generally synthesized by corrosion techniques, either by chemical corrosion in acidic solutions [3–7] or by electrochemical etching at anodic potentials [8–10]. Using the latter method, electrochemical dealloying, nanoporous gold (NPG) materials with ligament sizes of less than 6 nm can be effectively fabricated [5,10]. Zielasek et al. [4] and Xu et al. [11] reported that nanoporous gold, prepared by the selective dissolution of Ag from a AuAg alloy, exhibits a remarkably high activity for CO oxidation with molecular oxygen at low temperatures, and recent experiments in our laboratory arrived at comparable conclusions [12–13]. In the meantime, high catalytic activities of NPG catalysts were reported also for other reactions, such as oxidative coupling of methanol [14], aerobic oxidation of alcohols [15], and oxidation of organosilanols [16].

Until recently, high activities for the CO oxidation over Au catalysts were only reported for gold nanoparticles of a few nanometers in diameter, which are supported on reducible metal oxides such as TiO_2_, CeO_2_ and Fe_2_O_3_ [17–24]. For these oxide-supported Au catalysts, various parameters, such as the gold particle size, the chemical state of the gold nanoparticles or the nature and morphology of the support material have been shown to influence the catalytic activity [19,21]. Especially the nature of the supporting material was found to be decisive for the catalytic activity, with high activities only for catalysts supported on active, reducible metal oxides and only very low activities for those based on inert, nonreducible metal oxides [19,25]. For this reason, the finding of high activities for unsupported NPG was somewhat unexpected, since contributions from a support material are not possible here. As a consequence, the physical origin of the catalytic activity of the unsupported NPG materials has been under debate ever since.

The absence of the oxide support, however, can also be considered as an advantage for the understanding of the mechanisms responsible for the catalytic activity of NPG catalysts, and hence the catalytic behavior of nanoscaled Au, without the complications brought about by the interactions between the support material and the Au nanoparticles and the direct involvement of the support material in the catalytic reaction. Accordingly, earlier studies aiming at the understanding of the reaction mechanism and the physical origin of the high catalytic activity of NPG materials discussed this mainly in terms of structural effects and related modifications in the local electronic properties [4–5]. In these studies, gold atoms with low coordination numbers at surface defect sites, e.g., steps, corners and kinks, whose concentration increases with decreasing Au particle size or, for NPG, Au ligament size, were proposed as active sites for the activation of molecular oxygen, which is the key step for gold-catalyzed oxidation reactions [26–27]. Furthermore, quantum size effects, which have been proposed by Chen and Goodman for oxide-supported Au catalysts [28], may also be relevant for NPG catalysts considering the nanometer size of the Au ligaments. More recently, residual amounts of the second, less noble metal in the Au alloy used for NPG formation, such as Ag [13,29–30], Cu [6] or Al [31], which cannot be fully removed during dealloying [29], were proposed to be responsible for the unexpected high catalytic activity of NPG catalysts [4,32–33]. This would agree also with earlier proposals for Ag-contaminated submicron-size Au nanoparticles [34] and highly active mesoporous oxide-supported AuAg catalysts [35–36]. From calculations based on density functional theory, Moskaleva et al. found a significant reduction of the barrier for O_2_ dissociation in the vicinity of Ag surface atoms in a bimetallic AuAg surface [37]. On the other hand, Haruta considered the NPG catalyst as an inversely supported gold catalyst, where the junction perimeters between gold and Ag_2_O accounts for the high catalytic activity. A similar bifunctional effect was proposed also for CO oxidation on oxide-supported AuCu catalysts [38]. A recent study reported a NPG(Cu) catalyst to be very active for CO oxidation as well, but did not provide any mechanistic explanations [6]. Hence, in these pictures the NPG materials can be considered either as a bimetallic or as a bifunctional catalyst, where the second metal modifies the electronic and therefore also chemical properties of the Au surface atoms, or where oxidation of the second metal leads to a local metal–oxide interface.

To clarify whether one of these effects is dominating, and if so which, it would be best to completely separate these two aspects by using NPG samples that are completely free of the second metal or to vary the structural properties of the nanostructured NPG material, such as surface area or crystallite/ligament size, at a constant (surface) content of the respective second metal. Both of these approaches are not possible from experimental reasons, since it is not possible to completely remove all of the less noble metal, such as Ag and Cu, from the starting alloy [10], and also since these impurities sensitively affect the structural properties of the resulting NPG materials [13]. Therefore, these aspects have to be explored in a more indirect way, e.g., by comparing the effect of different residual metals on the catalytic properties of the NPG materials over a wide range of structural parameters and (surface) content of the respective second metal.

This is the topic of the present paper, in which we compare results on the low-temperature CO oxidation behavior of NPG catalysts with two different residual materials, Ag and Cu, and with different (surface and bulk) contents of these residues, focusing on differences and similarities in the role of these metals on the reaction behavior. A more extensive account of the experimental procedures and of the results on the Ag-containing NPG catalysts can be found in [12]. By applying different potentials during the electrochemical dealloying of AuAg and AuCu alloys, a series of NPG(Ag) and NPG(Cu) catalysts with varying Au ligament sizes and residual Ag(Cu) contents were obtained. The resulting samples were characterized by scanning electron microscopy (SEM), powder X-ray diffraction (XRD) and X-ray photoelectron spectroscopy (XPS) with respect to their morphology, structure and chemical composition. In order to unravel possible structure–activity relationships, the measurements were performed both on the fresh NPG samples and after reaction at atmospheric pressure for 1000 min. Measurements of the catalytic activity for CO oxidation of the NPG catalysts were performed in a conventional microreactor at atmospheric pressure and in a temporal analysis of products (TAP) reactor under ultrahigh vacuum (UHV) conditions. Further information on similarities and differences of the two different types of NPG catalysts in the reaction mechanism was obtained from multipulse TAP reactor measurements, which give access to the ability of the NPG catalysts to activate molecular O_2_ and to deposit stable adsorbed active oxygen species on the surface. The results are discussed in a comprehensive picture, in an attempt to combine both structural effects (“structure-activity correlations”) and the role of the second, less noble metal.

## Experimental

### Nanoporous gold sample preparation

**NPG(Ag):** Nanoporous gold samples were prepared by electrochemical etching (dealloying) of an Ag–Au alloy, as reported previously [13,39–40]. In short, the master alloy Ag_75_Au_25_ (atom %) was prepared by arc melting of high purity Au and Ag wires (Au 99.9985% and Ag 99.99%, Chempur) and subsequent homogenization at 950 °C for 70 h (sealed in a quartz tube). The ingots were rolled to 0.5 mm thick disks and annealed at 500 °C for two hours in vacuum for recovery. Electrochemical etching (dealloying) was performed in 1 M HClO_4_ aqueous solution under potentiostatic control. In order to obtain samples with different amounts of residual Ag, different potentials were applied for the dealloying procedure: 1.280 V_SHE_ (NPG(Ag)-1), 1.330 V_SHE_ (NPG(Ag)-2), and 1.380 V_SHE_ (NPG(Ag)-3 and NPG(Ag)-4). The potential was measured by a pseudo Ag/AgCl reference electrode (in 1 M HClO_4_), but is quoted versus that of a standard hydrogen electrode (SHE), whose potential is 0.53 V lower than that of the Ag/AgCl electrode. Dealloying was stopped when the current fell to 10 μA. After dealloying, the electrolyte was replaced by fresh base electrolyte and a higher potential (either 1.530 V_SHE_ for NPG(Ag)-1, NPG(Ag)-2 and NPG(Ag)-3 or 1.480 V_SHE_ for NPG(Ag)-4) was applied to the sample for about one hour to further remove the Ag ions, which had remained in the pore channels (see below in Table 1). Subsequently, the nanoporous gold samples were repeatedly rinsed with pure water for cleaning. The NPG(Ag) disks were dried, crushed and gently ground into powder before use, with grain sizes in the range of 20 to 100 μm.

**NPG(Cu):** Similar to the NPG(Ag) catalysts, a series of NPG samples containing varying amounts of residual Cu was prepared by electrochemical dealloying of a Au_25_Cu_75_ (atom %) alloy. The master alloy was prepared by arc melting of high purity Au and Cu wires (Au 99.995% and Cu 99.99%, Chempur) and subsequent homogenization at 900 °C for 4 days (sealed in a quartz tube), followed by quenching in water. The ingot was rolled to 0.2 mm thickness and annealed at 600 °C for 2 h in vacuum for recovery. Dealloying was performed in 1 M HClO_4_ aqueous solution (using deionized ultrapure water with electrical resistivity of 18.2 MΩ cm^2^/cm), at potentials of 1.430, 1.480 and 1.530 V_SHE_ (Ag/AgCl reference electrode placed directly in the 1 M HClO_4_ electrolyte close to the sample) for approximately one day. To minimize copper contamination in the sample compartment, the coiled-Cu wire counter electrode (CE) and the reference electrode (RE) were separated from the main cell by placing them in a tube filled with the same solution and mounted with its opening close to the sample. Also in this case, dealloying was stopped when the current fell to 10 μA. Further treatment of the NPG(Cu) samples resembled that for NPG(Ag) described above.

### Catalyst characterization

As mentioned previously [13], it is not possible to determine the surface area of the NPG samples under the normal conditions of a BET measurement, since its microstructure is thereby destroyed. For this reason we determined the surface area of the as-prepared NPG catalysts by the electrochemical capacitance ratio method [41].

X-ray diffraction (XRD) provided a separate, independent approach towards the structure of the NPG samples. The measurements were performed on a PANalytical MPD PRO instrument by using Cu Kα radiation (λ = 0.154 nm). The structural coherency was evaluated through the width of the Au(111) diffraction peaks by means of the Scherrer equation, *D*_Scherrer_ = (*Κ*·λ)/(β*·*cos θ), where *K=* 0.89 is the Scherrer constant, λ the wavelength of the X-rays, and β the FWHM. In the idealized case of nanoscale crystallites that are free of strain, *D*_Scherrer_ approximates the crystal size. NPG consists of crystals with a size on the order of 10 to 100 µm, which are porous on the much smaller nanometer scale [39,42]. The width of the diffraction peaks and the defect structure of the material [39,42] imply that the contribution of lattice defects/lattice strain to the reflection broadening may not be neglected. Overall, the Scherrer equation is therefore not an appropriate way of determining the ligament size. Nonetheless, the width of the Bragg reflections indicates a loss of crystalline coherence on a length-scale on the order of *D*_Scherrer_, and in this sense, the broadening of the XRD profiles may be discussed as an indicator of structure size. Moreover, if one assumes idealized, cylindrical ligaments with diameter *D,* the mass-specific surface areas (α) of the NPG samples can be calculated from the ligament size by the relation α = 4/(ρ·*D*) [40], where ρ is the mass density of gold (19.32 g·cm^−3^). Our results even indicate a quantitative correlation between the specific surface area as measured by the capacitance method and the value inferred by using *D*_Scherrer_ as the ligament size and assuming cylindrical ligaments (see below, Results and Discussion, subsection 1.1). Keeping in mind that this is an empirical relation, we will use the term *apparent ligament size* for the value derived from *D*_Scherrer_, and use this for the characterization of samples after the catalytic reaction, which cannot be characterized by the capacitance ratio method.

For further characterization of the samples, scanning electron microscopy (SEM) and energy dispersive X-ray spectroscopy (EDX) measurements were performed to determine the surface morphology, microstructure and the bulk concentration of Ag/Cu, respectively. In order to determine the surface concentration of residual Ag or Cu, respectively, X-ray photoelectron (XP) spectra were recorded on the NPG powder samples by using a PHI 5800 ESCA system (Physical Electronics) with monochromatic Al Kα radiation for excitation. The binding energies of the Au(4f), Ag(3d) and Cu(2p) states were calibrated with respect to the C(1s) peak at 284.6 eV. The Au, Ag and Cu surface concentrations were calculated from the measured intensities of the Au(4f), Ag(3d) and Cu(2p) signals, respectively, by using tabulated sensitivity factors. It should be noted that this procedure assumes a constant composition of the top few layers. Repeated XPS measurements on the same samples and also on samples before grinding them into powder revealed that the error for the resulting surface Ag content is less than 5% (relative).

#### Catalytic activities: Flow reactor measurements

In a similar manner to the measurements in [12–13], the catalytic activities of the NPG catalysts for CO oxidation were measured in a microreactor with a length of 300 mm, an outer diameter of 6 mm, and an inner diameter of 4 mm at atmospheric pressure and a reaction temperature of 30 °C, without applying any pretreatment prior to the measurements. The catalysts were diluted with α-Al_2_O_3_ in order to obtain differential reaction conditions, with reactant conversions of below 15% throughout the measurements. The temperature of the catalyst bed was measured by a thermocouple attached to the outer wall of the reactor, in the central area of the catalyst bed. The flow rate of the reactant gas was 60 NmL·min^−1^ (1% CO, 1% O_2_, rest N_2_), and both influent and effluent gases were analyzed by on-line gas chromatography (Chrompack CP9001). For further details of the setup and the evaluation see [19,43].

#### Catalytic activities: TAP reactor measurements

The pulse experiments were carried out in a home-built TAP reactor [44], which is largely based on the TAP-2 approach of Gleaves et al. [45], using a similar approach as was described previously in [12–13]. In short, piezoelectric pulse valves were used to generate gas pulses of typically ~1 × 10^16^ molecules per pulse. For all measurements presented, these pulses contained 50% Ar as an internal standard to enable quantitative evaluation on an absolute scale. The gas pulses were directed into a quartz-tube microreactor with a length of 90 mm, an outer diameter of 6 mm, and an inner diameter of 4 mm. The catalyst bed was located in its central part, fixed by two stainless steel sieves (Haver & Boecker OHG, transmission 25%). For all measurements, we used a three-zone catalyst bed containing 2 mg of NPG catalyst diluted with 20 mg SiO_2_ as the central zone and two layers of SiO_2_ as the outer zones (total mass 150 mg). All pulse experiments were performed at 30 °C reaction temperature. Also here, the catalyst was used as received, with no additional pretreatment prior to the measurements. After passing through the reactor, the gas pulses were analyzed by a quadrupole mass spectrometer (QMG 700, Pfeiffer) located behind the reactor tube in the analysis chamber. The consumption of CO and O_2_ in the respective pulses was calculated from the missing mass spectrometric intensity in the pulses compared to the intensity after surface saturation, which is equivalent to the initial intensity. The formation of CO_2_ could be determined directly from the CO_2_ pulse intensity. Additionally, (pre)treatment of the catalysts at higher (atmospheric) pressure is possible by separating the reactor from the ultrahigh vacuum (UHV) system by a differentially pumped gate valve and connecting it directly to an adjustable roughing pump.

For testing the catalytic activity for CO oxidation in the TAP reactor, the samples were exposed to simultaneous pulses of CO/Ar and O_2_/Ar, with a CO/O_2_ ratio of 1:1, i.e., an excess of oxygen relative to stoichiometric reaction conditions. Prior to these measurements it was checked that the gas mixing unit and the gas pipes containing the reaction mixture as well as the reactor and the dilution materials were inert; no conversion of CO or O_2_ was found under these conditions in control experiments. To identify stable adsorbed surface species on the catalyst surface before and after the reaction, we also performed temperature-programmed desorption (TPD) measurements in the TAP reactor. During these measurements, the catalysts were heated from 30 to 600 °C at a heating rate of 25 °C·min^−1^. The gaseous desorption/decomposition products are transported by diffusion into the analysis chamber, where they are detected by the mass spectrometer.

The ability to activate O_2_, given by the amount of stable adsorbed active oxygen that can be reversibly deposited from interaction with O_2_ and reacted away by CO pulses (oxygen storage capacity, OSC), was also determined by TAP reactor measurements. Here, the NPG samples were first exposed to O_2_ in a continuous flow of 10% O_2_/N_2_ with a gas flow of 20 NmL·min^−1^ at atmospheric pressure (30 min at 30 °C); afterwards the amount of stable adsorbed oxygen species active for CO oxidation was titrated by a sequence of CO/Ar pulses under vacuum conditions. The formation of CO_2_ originating from the reaction of CO with adsorbed oxygen could be determined directly from the CO_2_ pulse intensity, and the OSC was calculated from the total amount of CO_2_ produced during a sequence of CO pulsing [12]. Details about the calibration of the mass spectrometric signals on an absolute scale can be found in [44]. Direct evaluation of the OSC by quantification of the O_2_ uptake of a reduced sample during O_2_ pulses, as performed for supported catalysts [23,25,46], was not possible due to the very low amount of oxygen uptake within single O_2_/Ar pulses. After the oxygen species were removed, the catalyst was exposed once again to a mixture of 10% O_2_/N_2_ at atmospheric pressure, and the process of oxygen deposition and reactive removal by CO was repeated at least three times for all samples in order to check the reversibility of the active oxygen formation.

## Results and Discussion

### 1 Structural and chemical characterization of the NPG catalysts

#### 1.1 NPG(Ag) catalysts

**Figure 1:**
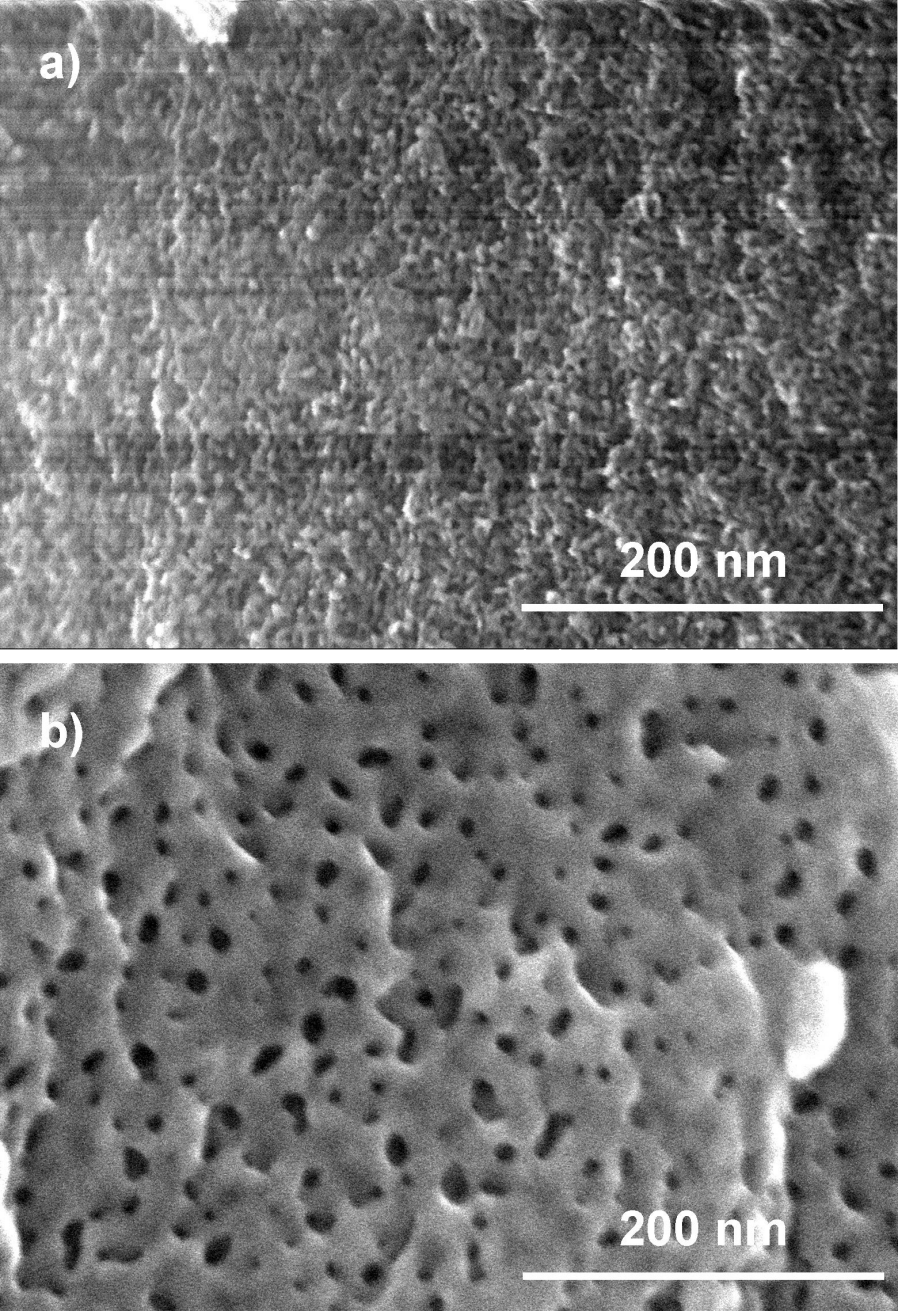
SEM images of the NPG(Ag)-4 catalyst (a) before and (b) after 1000 min on stream.

**Figure 2:**
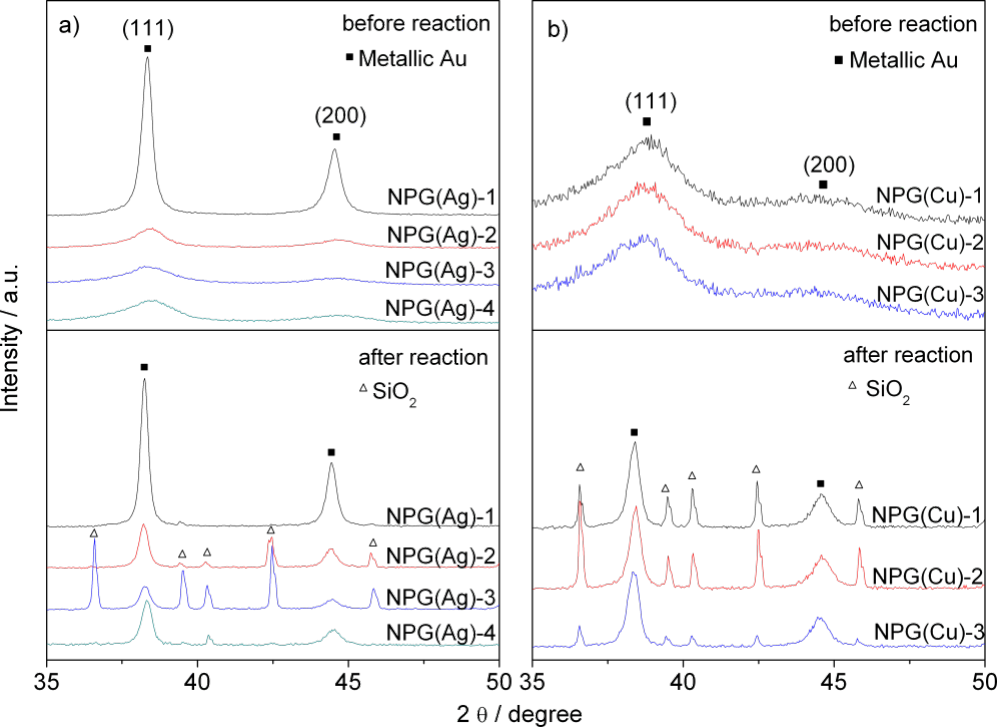
XRD patterns of various NPG catalysts before and after 1000 min on stream: (a) NPG(Ag) and (b) NPG(Cu).

**As-prepared samples:** The morphology of the NPG materials was characterized by SEM imaging. Figure 1 displays representative SEM images of the NPG(Ag)-4 sample before and after the catalytic reaction. The fresh sample has a foamlike morphology with ligament diameters of below 10 nm (Figure 1a). More detailed structural features, however, are not discernible due to the limited resolution of the SEM image. To gain further information on the structural properties of the bulk phase, XRD measurements were performed on these samples (Figure 2a). Considering the discussion in the Experimental subsection „Catalyst characterization“, the broad diffraction peaks of metallic Au for the three fresh samples, NPG(Ag)-2 to NPG(Ag)-4, indicate that the crystallites are significantly strained, possibly due to a large density of lattice dislocations. On NPG(Ag)-1, the diffraction peaks are significantly sharper, indicating a lower defect density. This goes along with a significantly lower specific surface area of sample NPG(Ag)-1 as determined by the capacitance ratio method (see below). In none of the samples could we detect diffraction peaks characteristic of the Ag-containing phases (metallic silver or silver oxides). Using the Scherrer equation in the sense described before, we determined apparent ligament sizes of 21, 6.4, 4.1 and 4.1 nm for the samples NPG(Ag)-1, NPG(Ag)-2, NPG(Ag)-3 and NPG(Ag)-4, respectively (Table 1).

**Table 1:** Details of the preparation parameters, physical properties and catalytic activity for CO oxidation at 30 °C for various NPG(Ag) catalysts (values taken from [13]).^a^

catalyst	NPG(Ag)-1	NPG(Ag)-2	NPG(Ag)-3	NPG(Ag)-4

potential of dealloying / V_SHE_	1.280	1.330	1.380	1.380
potential of cleaning / V_SHE_	1.530	1.530	1.530	1.480
average crystallite size *D* / nm^b^	21 (28)	6.4 (21)	4.1 (23)	4.1 (21)
surface area / m^2^·g^−1 c^	≈10	63	59	75
surface area / m^2^·g^−1 d^	10 (7)	31 (10)	51 (9)	50 (10)
bulk Ag / atom %^e^	4.5	13.2	14.4	14.6
surface Ag / atom %^f^	5.4 (16.6)	9.5 (30.0)	10.5 (24.7)	18.6 (25.3)
surface Ag atoms / 10^20^·g_cat_^−1^	0.06 (0.13)	0.69 (0.34)	0.71 (0.26)	1.6 (0.29)
OSC–stable / 10^19^ O atoms·g_Au_^−1^	0.03	1.2	0.54	0.86
*r*_Au_ / 10^−5^ mol·s^−1^·g_Au_^−1^	0.4 (0.3)	12.4 (9.2)	3.4 (3.6)	18.2 (6.2)

^a^The data presented in parentheses are obtained from the samples after 1000 min on stream. ^b^Estimated from Au(111) diffraction peaks by using the Scherrer equation. ^c^Surface areas of the fresh samples measured by the capacitance ratio method. ^d^Estimated by assuming idealized, cylindrical ligaments with diameter *D*. ^e^Measured by SEM-EDX. ^f^Measured by XPS.

**Figure 3:**
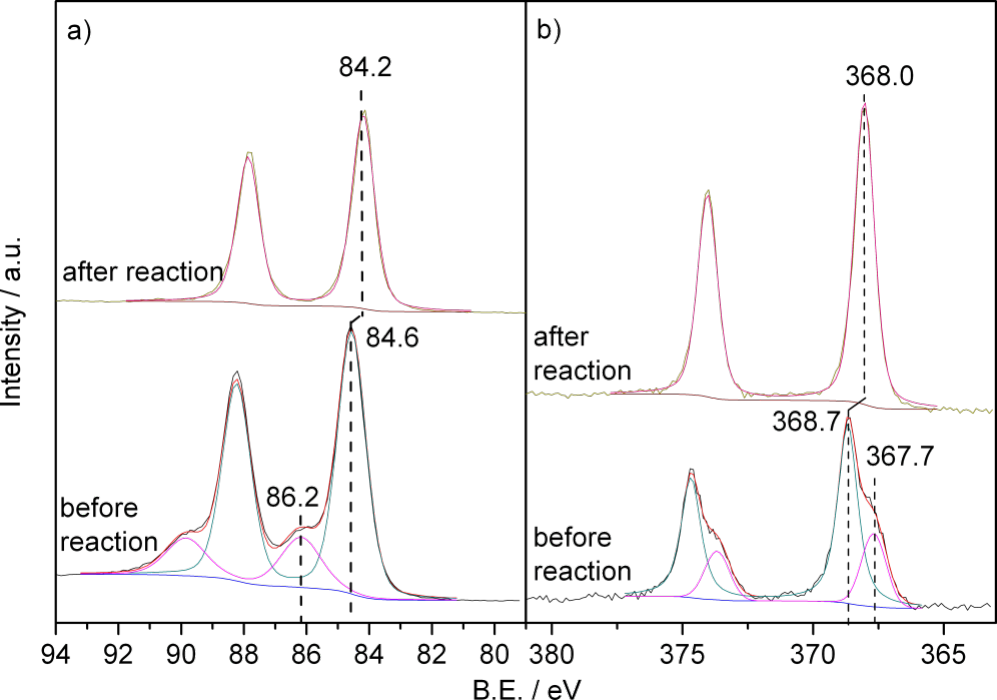
Au(4f) (a) and Ag(3d) (b) XP spectra of the NPG(Ag)-3 catalyst before and after 1000 min on stream.

Figure 3 shows XP detail spectra of the Au(4f) and Ag(3d) regions of an as-prepared NPG(Ag) sample. For brevity, we here show only those of the NPG(Ag)-3 sample; the corresponding spectra for the other three samples are given in Supporting Information File 1 (Figure S1). The characteristic parameters evaluated from these XP spectra are summarized in Table 2. The Au(4f) spectrum contains two sets of distinct peaks, with the Au(4f_7/2_) peaks at 84.6 and 86.2 eV. The binding energy (BE) of the main peak is slightly higher than that for metallic Au^0^ (84.0 eV) [47–48]. The shoulder at higher BE can be assigned to Au^3+^ (Au_2_O_3_) species, following previous assignments [48]; it contributes about 21% to the total Au(4f) intensity based on a deconvolution of the peaks. Similar spectra were also obtained on the NPG(Ag)-2 and NPG(Ag)-4 samples, with contributions of 11% and 12% from Au^3+^ species, respectively. In contrast, no oxidic species were detected on the NPG(Ag)-1 sample. The Ag(3d) XP spectra were also recorded to check the existence of residual Ag and its chemical state. As shown in Figure 3b, the as-prepared NPG(Ag)-3 sample contains two distinct components of the Ag(3d_5/2_) peak with BEs of 368.7 eV and 367.7 eV, respectively. At first glance, the second peak seems to be more characteristic for metallic Ag^0^ [35], while the high BE of the first peak does not fit with literature reports [49]. Considering, however, that the final-state effects will lead to a similar up-shift of the BEs as discussed for the Au(4f) peaks, we assign the Ag(3d_5/2_) peak at 368.7 eV to metallic Ag, up-shifted by final-state effects, while the 367.7 eV peak is attributed to a similarly up-shifted Ag(3d_5/2_) peak related to AgO [49], with a contribution of about 28% to the total Ag(3d) intensity. The corresponding fractions of silver oxide species on the NPG(Ag)-2 and NPG(Ag)-4 catalysts were 22% and 26%, respectively, while only metallic Ag was found on the NPG(Ag)-1 sample.

**Table 2:** XPS results of various NPG(Ag) samples before and after reaction.

catalyst	B.E. of Au(4f_7/2_) / eV	Au^3+^ / %	B.E. of Ag(3d_5/2_) / eV	AgO / %	surface Ag / atom %

NPG(Ag)-1 fresh	84.5	0	368.3/—	0	5.4
NPG(Ag)-1 used	84.4	0	368.2/—	—	16.6
NPG(Ag)-2 fresh	84.5	11	368.6/367.8	22	9.5
NPG(Ag)-2 used	84.3	0	368.1/—	—	30.0
NPG(Ag)-3 fresh	84.6	21	368.7/367.7	28	10.5
NPG(Ag)-3 used	84.2	0	368.0/—	—	24.7
NPG(Ag)-4 fresh	84.5	12	368.5/367.7	26	18.6
NPG(Ag)-4 used	84.3	0	368.2/—	—	25.3

Table 1 lists the Ag contents (both bulk content (EDX) and content in the near-surface region (XPS)) and the specific surface areas of the different NPG(Ag) samples. Obviously, raising the dealloying potential leads to an increase of the residual (bulk) Ag content in the NPG(Ag) samples, though with a nonlinear relationship. A potential increase from 1.280 V_SHE_ to 1.330 V_SHE_ results in a more significant Ag retention in the bulk phase (4.5 and 13.2 atom % Ag for NPG(Ag)-1 and NPG(Ag)-2, respectively), while for a further potential increase to 1.380 V_SHE_ the changes are less pronounced, with 14.4 and 14.6 atom % Ag for NPG(Ag)-3 and NPG(Ag)-4, respectively. The surface Ag contents show a similar dependence on the dealloying potential as observed in the bulk phase, with surface Ag contents of 5.4, 9.5, 10.5 and 18.6 atom % Ag for NPG(Ag)-1 to NPG(Ag)-4, respectively (see Table 1). The surface Ag content of the sample NPG(Ag)-4, however, is remarkable, since it is significantly higher compared to that of the NPG(Ag)-3 sample, although it has a comparable bulk Ag content and the same dealloying potential was applied. Most probably, this discrepancy is caused by the lower potential used during the following cleaning procedure. The present data agrees well with a recent study of Ag retention during electrochemical dealloying of AuAg at different pH values by Liu et al. [50]. Those authors also showed that it is possible to obtain samples with significantly different surface Ag content by varying the dealloying potential (at constant dealloying time and sample size) and the subsequent cleaning procedure [50].

The higher dealloying potential not only caused an increase in the Ag content (bulk and near-surface Ag contents) of the NPG(Ag) samples, but also in the surface areas measured directly by the capacitance ratio method. The latter changes are particularly obvious at lower potentials, from 1.280 V_SHE_ to 1.330 V_SHE_. As described in the Experimental section (subsection „Catalyst characterization“), we also estimated the surface areas from the XRD data, assuming idealized, cylindrical ligaments and using the apparent ligament size. The results are comparable to the data measured directly for the samples NPG(Ag)-1 (10 m^2^·g_cat_^−1^) and NPG(Ag)-3 (51 m^2^·g_cat_^−1^), whereas for the samples NPG(Ag)-2 (31 versus 63 m^2^·g_cat_^−1^) and NPG(Ag)-4 (50 versus 75 m^2^·g_cat_^−1^) the directly measured surface areas were higher than those derived from the XRD data (see Table 1). This discrepancy underlines the dangers in applying the Scherrer equation to materials with nanoscale pores in micron-scale crystals. In fact, previous TEM investigations revealed a large number of lattice defects in NPG, specifically when the dealloying conditions are set to obtain a very small ligament size [39]. In order to correct for differences/changes in the Ag surface content in the respective NPG samples, we also calculated the total amount of surface Ag atoms per gram of catalyst (see Table 1), assuming a homogeneous vertical distribution of the Ag atoms in the surface regime analyzed by XPS. This was done by multiplying the surface Ag content with the total number of surface atoms, which was determined from the surface area by assuming a surface density of 1.15 × 10^15^ atoms cm^−2^. The resulting values are given in Table 1.

**Catalysts after reaction:** Since the NPG(Ag) catalysts deactivated to different extents during 1000 min on stream (see section 2 "Catalytic activities"), we followed the structural changes during the activity measurements in order to detect possible structure-activity correlations. The NPG(Ag) catalysts were retrieved after reaction for 1000 min and subsequently characterized again. A representative SEM image taken from the NPG(Ag)-4 sample after reaction showed a distinct coarsening of the porous surface structure (see Figure 1b). This is confirmed also by the XRD patterns (see Figure 2a), where exposure to the reaction gas mixture caused a considerable sharpening of the diffraction peaks for all NPG(Ag) catalysts. Hence, restructuring occured also in the bulk region. These peak widths correspond to apparent ligament sizes of 28, 21, 23 and 21 nm for NPG(Ag)-1 to NPG(Ag)-4 after reaction, respectively. A similar reaction-induced ligament size growth for nanoporous gold during CO oxidation (25 h, 30 °C, 66.7 mL·min^−1^: 1% CO + 10% O_2_ + 89% N_2_,) was reported also by Xu et al. [5]. Note that since SiO_2_ was used as a dilution material during reaction, its sharp peaks can also be identified in the XRD patterns after reaction.

After exposure to the reaction conditions for 1000 min, the higher BE Au(4f_7/2_) XPS shoulder completely disappeared, and the main Au(4f_7/2_) peak shifted to a lower BE of 84.2 eV (see Figure 3a). This result clearly indicates that the surface Au^3+^ oxide species are reduced under the reaction conditions. The down-shift of the main peaks can originate from the reduction of slightly oxidized Au species (e.g., Au^+^ with a BE 0.6 eV higher than that of Au^0^ [51–52]) and/or from the significant growth of the ligament size during reaction (from ca. 4.1 nm to ca. 23 nm, as described above). It has been reported that BE shifts of 0.5–0.9 eV relative to the bulk Au^0^ value could be obtained as final-state effects in small Au clusters [53–54]. With increasing particle size, the Au(4f) peak would shift back to the metallic Au^0^ position. However, in view of the relatively large average Au ligament size (apparent ligament size 4.1 nm) for the fresh sample, larger contributions from final-state effects are considered to be not very likely. Therefore, we favor an explanation that relates the higher BE of the peak at 84.6 eV (before reaction) mainly to positively charged gold species (Au^δ+^), which are discharged during the reaction. The exact charge of this Au^δ+^ species, however, cannot be determined from these measurements. Going to the Ag(3d) signals, we find that after reaction for 1000 min the doublet of the Ag(3d) XPS peaks detected on the fresh samples for NPG(Ag)-2 to NPG(Ag)-4 is replaced by a new peak with an intermediate BE of 368.0 eV (see Figure 3b). This corresponds to the normal BE of metallic Ag^0^, without further shifts introduced by final state effects [35,49]. Thus, similar to Au, oxidized Ag surface species are reduced during the reaction.

Table 1 also contains the chemical compositions as well as the surface areas of the various NPG(Ag) samples after reaction. Unlike the fresh samples, the surface area of the samples after reaction could not be measured by the capacitance ratio method, since the amounts of NPG(Ag) catalyst used for reaction are very low (2 mg) and the samples were crushed before use. Therefore, the surface areas of the used NPG(Ag) catalysts were estimated from the apparent ligament sizes, as described above. Here we should keep in mind the approximate character of the X-ray diffraction analysis and the deviations between the surface areas determined by different methods, which are expected also for the surface areas determined after reaction. Deviations in the surface areas will directly affect the number of Ag or Au surface atoms per gram of catalyst. Interestingly, the distinct differences in the ligament sizes of the fresh NPG(Ag) catalysts decay significantly during reaction for 1000 min, resulting in much more similar values. Hence, the structure of the NPG(Ag) changes considerably during the reaction, and these changes are much more pronounced for the samples with initially smaller ligaments (NPG(Ag)-2 to NPG(Ag)-4). This finding agrees well with those in previous reports [11].

Based on the XPS measurements, the reaction also caused a significant surface Ag enrichment for all four NPG(Ag) samples. The surface Ag content increased by a factor of 3 to 16.6 and 30.0 atom % for NPG(Ag)-1 and NPG(Ag)-2, respectively, compared with that before reaction. For the samples NPG(Ag)-3 and NPG(Ag)-4 the Ag surface enrichment was lower, and increased only by a factor of 2.4 to 24.7 atom % and by a factor of 1.4 to 25.3 atom %, respectively. Surface enrichment of Ag in bimetallic AuAg systems has previously been detected both experimentally [35,55] and in theoretical simulations [56–57]. For example, in a study of CO oxidation over Au–Ag/TiO_2_ catalysts, Sandoval et al. [55] observed a surface Ag enrichment upon thermal treatment at high temperatures. The extent of Ag enrichment on the surface of AuAg alloys depends sensitively on temperature, composition and particle size [56]. From a thermodynamic point of view, Ag prefers either to be on the surface or to form interfacial alloys with Au, while Au prefers to segregate at the core due to the lower surface energy of Ag compared to Au in the absence of adsorbates [56,58]. This may be modified by strongly adsorbing species. The number of surface Ag atoms after reaction was also calculated to compare with the data obtained on the fresh samples (see Table 1).

#### 1.2 NPG(Cu) catalysts

For the Cu-containing NPG catalysts, we employed an approach similar to the preparation of NPG(Ag) catalysts to prepare a series of NPG(Cu) samples with different residual Cu contents by using different dealloying potentials in the range of 1.430–1.530 V_SHE_ during the etching process. This potential range was chosen since initial experiments had shown that highly active NPG(Cu) samples with small ligament sizes (see below), which are comparable to those of the samples NPG(Ag)-2 to NPG(Ag)-4, could only be obtained when the dealloying was performed at potentials higher than 1.380 V_SHE_.

**As-prepared samples:** The characterization results presented in Table 3 show that, within the range investigated, the dealloying potential had no significant influence on the surface area (58, 68 and 49 m^2^·g^−1^ for NPG(Cu)-1 to NPG(Cu)-3, respectively). The rather broad XRD diffraction peaks (see Figure 2b), which are similar for all samples, indicate that there are also no distinct differences in the structural properties; the apparent mean ligament sizes were calculated to be 3.5, 3.3 and 3.3 nm, with the corresponding estimated surface areas of 60, 63 and 62 m^2^·g^−1^ for the samples NPG(Cu)-1, NPG(Cu)-2 and NPG(Cu)-3, respectively (see Table 3). The calculated surface areas fit well with the ones measured directly via the capacitance ratio method.

**Table 3:** Details on the preparation parameters, physical properties and catalytic activity for the CO oxidation at 30 °C for various NPG(Cu) catalysts.^a^

catalyst	NPG(Cu)-1	NPG(Cu)-2	NPG(Cu)-3

potential of dealloying / V_SHE_	1.430	1.480	1.530
potential of cleaning / V_SHE_	1.530	1.530	1.530
average crystallite size *D* / nm^b^	3.5 (24)	3.3 (23)	3.3 (22)
surface area / m^2^·g^−1 c^	58	68	49
surface area / m^2^·g^−1 d^	60 (9)	63 (9)	62 (10)
bulk Cu / atom %^e^	6.8	6.0	4.5
surface Cu / atom %^f^	3.4 (5.3)	3.7 (5.7)	4.1 (5.4)
amount of surface Au atoms / 10^20^·g_cat_^−1^	6.6 (0.96)	7.0 (0.96)	6.9 (1.02)
amount of surface Cu atoms / 10^19^·g_cat_^−1^	2.3 (0.54)	2.7 (0. 57)	3.0 (0.58)
OSC–stable / 10^19^ O atoms·g_Au_^−1^	0.07	0.13	0.23
*r*_Au_ / 10^−5^ mol·s^−1^ g_Au_^−1^	0.9 (1.5)	2.4 (2.8)	2.6 (3.5)

^a^The data presented in parentheses are obtained from the samples after 1000 min on stream. ^b^Estimated from Au(111) diffraction peaks by using the Scherrer equation. ^c^Surface areas of the fresh samples measured by the capacitance ratio method. ^d^Estimated by assuming idealized, cylindrical ligaments with diameter *D*. ^e^Measured by SEM-EDX. ^f^Measured by XPS.

**Figure 4:**
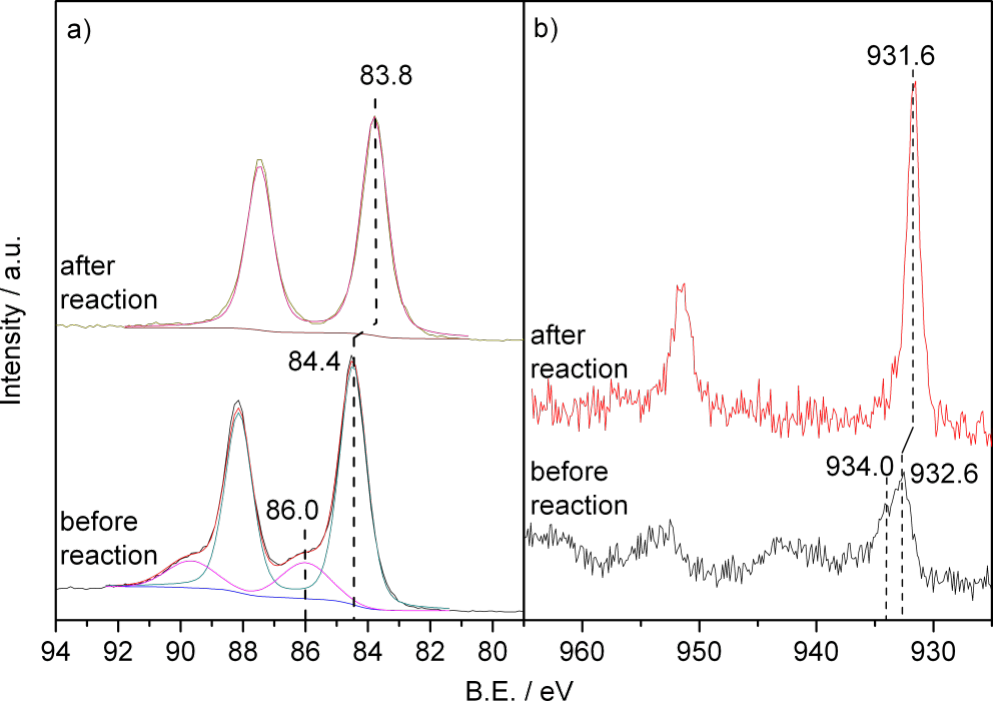
Au(4f) (a) and Cu(2p) (b) XP spectra of the NPG(Cu)-2 catalyst before and after 1000 min on stream.

Au(4f) and Cu(2p) XP spectra recorded on the NPG(Cu)-2 sample, which are representative of all three NPG(Cu) samples, are presented in Figure 4. The parameters evaluated from the XP spectra are summarized in Table 4. Similar to the Ag-containing NPG samples, two Au(4f_7/2_) peaks with BEs of 84.4 and 86.0 eV were detected on all three samples investigated, with the fraction of the Au^3+^ oxide species (86.0 eV) being 22, 19 and 24% for NPG(Cu)-1 to NPG(Cu)-3, respectively. The main peak of the Cu(2p) spectra (Figure 4b) consists of two components at ≈932.6 and ≈934.0 eV, accompanied by a distinct satellite peak at ≈942 eV. According to the literature [59–61], the peak at ≈934.0 eV together with the satellite peak is characteristic of Cu^2+^ oxide species, whereas the peak at ≈932.6 eV may arise from copper oxide species with lower oxidation states, most likely Cu_2_O. An unambiguous assignment, however, requires further information, e.g., from the corresponding Auger spectra [60]. The elemental analysis showed an increasing surface Cu content with increasing dealloying potential, i.e., 3.4, 3.7 and 4.1 atom % for samples NPG(Cu)-1 to NPG(Cu)-3, respectively. These values are somewhat lower than the corresponding Cu contents in the bulk phase determined by EDX (6.8, 6.0 and 4.5 atom % for NPG(Cu)-1 to NPG(Cu)-3, respectively, see Table 3). This result differs from the trends for the fresh NPG(Ag) samples, where two of the catalysts had a lower and two a higher surface Ag content than the bulk (see Table 1). The physical origin for this discrepancy, however, is not clear yet.

**Table 4:** XPS results of various NPG(Cu) samples before and after reaction.

catalyst	B.E. of Au(4f_7/2_) / eV	Au^3+^ / %	B.E. of Cu(2p_3/2_) / eV	surface Cu / atom %

NPG(Cu)-1 fresh	84.5	22	934.1/932.7	3.4
NPG(Cu)-1 used	84.7	0	932.7	5.3
NPG(Cu)-2 fresh	84.4	19	934.0/932.6	3.7
NPG(Cu)-2 used	83.8	0	931.6	5.7
NPG(Cu)-3 fresh	84.5	24	934.2/932.7	4.1
NPG(Cu)-3 used	83.5	0	931.4	5.4

**Samples after reaction:** The diffractograms of the used catalysts (Figure 2b) show a significant sharpening of the diffraction peaks for all three samples after reaction for 1000 min, pointing to a pronounced coarsening of the bulk structure. The apparent ligament sizes were determined to be 24, 23 and 22 nm for NPG(Cu)-1 to NPG(Cu)-3 (see Table 3), respectively, close to the values obtained for the NPG(Ag) samples after reaction. The surface areas of these samples are calculated to be 9, 9 and 10 m^2^·g^-1^. XP spectra recorded after 1000 min on stream resolved only metallic Au surface species on all three samples, with the BE of Au(4f_7/2_) down-shifted to ≈83.8 eV for NPG(Cu)-2 (see Figure 4) and NPG(Cu)-3, again comparable to the findings for NPG(Ag) samples, while it stays almost constant for NPG(Cu)-1. For the surface Cu species, the satellite peaks disappeared after exposure to the reaction conditions, and the main Cu(2p) peak shifted to lower BE, to ≈931.6 eV for all NPG(Cu) samples, indicating the formation of metallic copper or Cu^+^ species [59,61–62]. Due to the presence of the dominant Au(4d_5/2_) peak at 335.0 eV, we cannot distinguish between these two species from their Cu LMM Auger peaks at 335.0 eV (Cu^0^) or 337.5 eV (Cu^+^), respectively (see also the discussion on the TPD results below, subsection 2.2). Finally, also in this case the XP spectra indicate a reaction-induced surface enrichment of Cu for all three NPG(Cu) samples, with 5.3, 5.7 and 5.4 atom % for the samples NPG(Cu)-1 to NPG(Cu)-3, respectively, although to a much lesser extent compared with the NPG(Ag) materials. This can be simply explained by the lower surface energy of Au compared to that of Cu [63].

### 2 Catalytic activities

#### 2.1 Catalytic activities in the microreactor

**NPG(Ag):** The catalytic activities of the NPG catalysts for CO oxidation were first determined in the commonly used approach, by kinetic measurements at atmospheric pressure in a microreactor under differential reaction conditions. Note that for the nanoporous Au material the kinetic parameters may be significantly influenced by the mass transport limitation by pore diffusion [29]. For the diluted powders of NPG samples used in this work, however, diffusion limitations are much less likely than for NPG disks used in a previous study [29]. In fact, by using the method described before [29], the Thiele modulus is estimated to be less than 3, where the influence of mass transport limitations is negligible.

**Figure 5:**
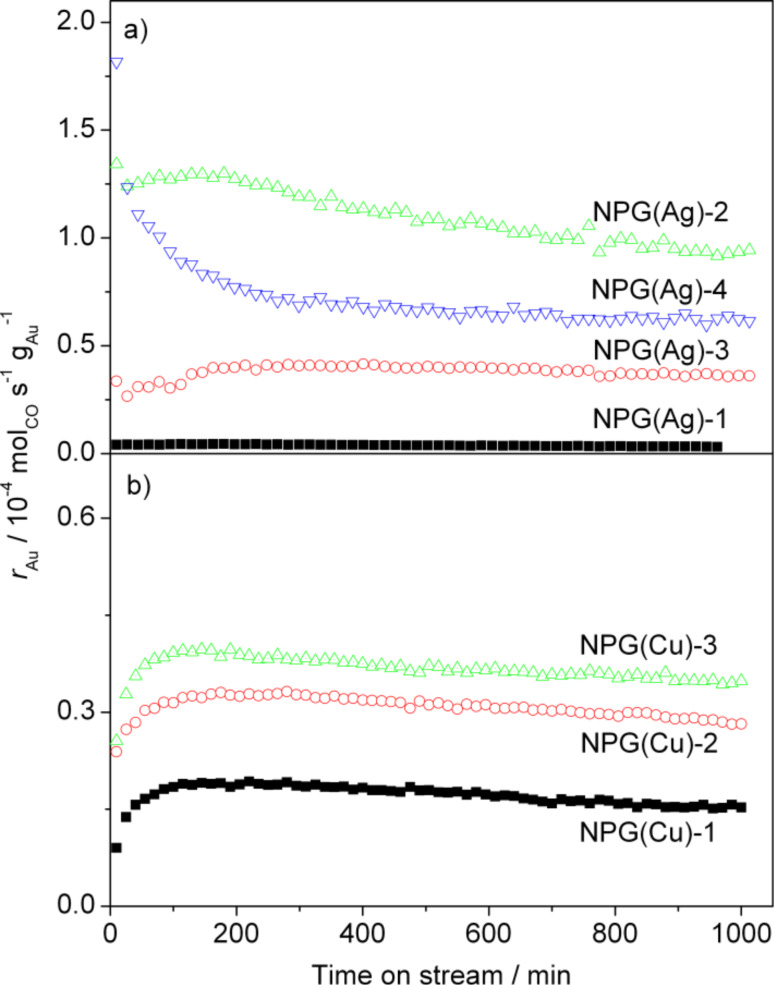
Temporal evolution of the catalytic activities of various (a) NPG(Ag) and (b) NPG(Cu) catalysts measured in a microreactor under differential reaction conditions. (Reaction temperature 30 °C, 60 NmL·min^−1^, 1% CO/1% O_2_/rest N_2_).

Figure 5a shows the CO oxidation activity of the four NPG(Ag) catalysts, in terms of Au mass-normalized reaction rates, and their temporal evolution in a standard reaction mixture (1% CO, 1% O_2_, rest N_2_) at 30 °C reaction temperature. Among the four samples investigated, the NPG(Ag)-4 catalyst showed the highest initial activity (1.8 × 10^−4^ mol_CO_·s^−1^·g_Au_^−1^), with a continuous decay over time during the first 200 min (60%), followed by an almost constant activity up to 1000 min on stream. This closely resembles the behavior of Au/TiO_2_ catalysts during CO oxidation [64–65]. In contrast, the other three NPG(Ag) catalysts exhibited a distinctly different reaction behavior. For these samples, the activity first increased steadily, for 2–3 h, and afterwards decayed by 20–30% (see Figure 5a). After 1000 min on stream, approximately steady-state activities are reached, which amount to 0.3, 9.2, 3.6, and 6.2 × 10^−5^ mol_CO_·s^−1^·g_Au_^−1^ for NPG(Ag)-1 to NPG(Ag)-4, respectively (see also Table 1). Note that despite the comparable surface areas under steady-state conditions, the NPG(Ag)-2 sample is more active than samples NPG(Ag)-3 and NPG(Ag)-4. The observation of an initial activation phase closely resembles findings by Wittstock et al., who reported an activation period of approximately 2–3 h on a NPG disk catalyst (ligament size 30–50 nm) for CO oxidation at 80 °C [29]. These authors related the activation to the removal of moisture in the pores or of contaminants stemming from the leaching process. The first suggestion, removal of moisture, appears to be unlikely considering the results of additional kinetic measurements performed in our laboratory, in which we used an additional drying pretreatment and dry feed gas [13]. These experiments revealed that while the activities are lower for all catalysts than under “normal” reaction conditions, the catalytic behavior is essentially unaltered (for details see [13]). Hence, the presence of moisture in the reaction gas mixture, even of the trace impurities as present in commercial gases and on the catalyst, raises the activity of NPG(Ag) catalysts, but does not change their typical reaction behavior with time on stream. The increase in activity even extends to higher moisture contents as evidenced by recent findings by Wittstock et al. [33] who observed that adding 10000 ppm water to the gas feed enhances the catalytic activity of NPG catalyst in CO oxidation, in their case by more than 100%.

**NPG(Cu):** Similar kinetic measurements performed on the three NPG(Cu) catalysts showed that the catalytic behavior closely resembles that of the NPG(Ag)-2 and NPG(Ag)-3 samples, showing an activation period of ca. 2 h followed by a continuous decay until reaching a quasi-steady-state after 1000 min on stream (see Figure 5b). Since the apparent ligament size and surface area for all Cu- and Ag-containing samples is almost the same after reaching the steady state, we use the stable activities to compare the NPG(Cu) and NPG(Ag) samples. Although the amount of Cu residues at the surface of NPG(Cu) catalysts is much lower compared to that of Ag in all NPG(Ag) catalysts (ca. 5.5% surface Cu content compared to 16.6–30.0% surface Ag content), the stable catalytic activities of the NPG(Cu) samples for CO oxidation reaction at 30 °C are even higher (NPG(Ag)-1) or comparable (NPG(Ag)-3) compared with those of the two less active NPG(Ag) catalysts (see Table 3). This indicates a higher efficiency of Cu in oxygen activation under the reaction conditions compared with that of Ag, considering the comparable particle sizes (≈20 nm) and assuming that Cu plays a similar role to that of Ag.

#### 2.2 TAP reactor measurements

In a second approach, the catalytic activities of the NPG catalysts for CO oxidation were examined in the TAP reactor under UHV conditions, starting with a fresh catalyst without any pretreatment.

**Figure 6:**
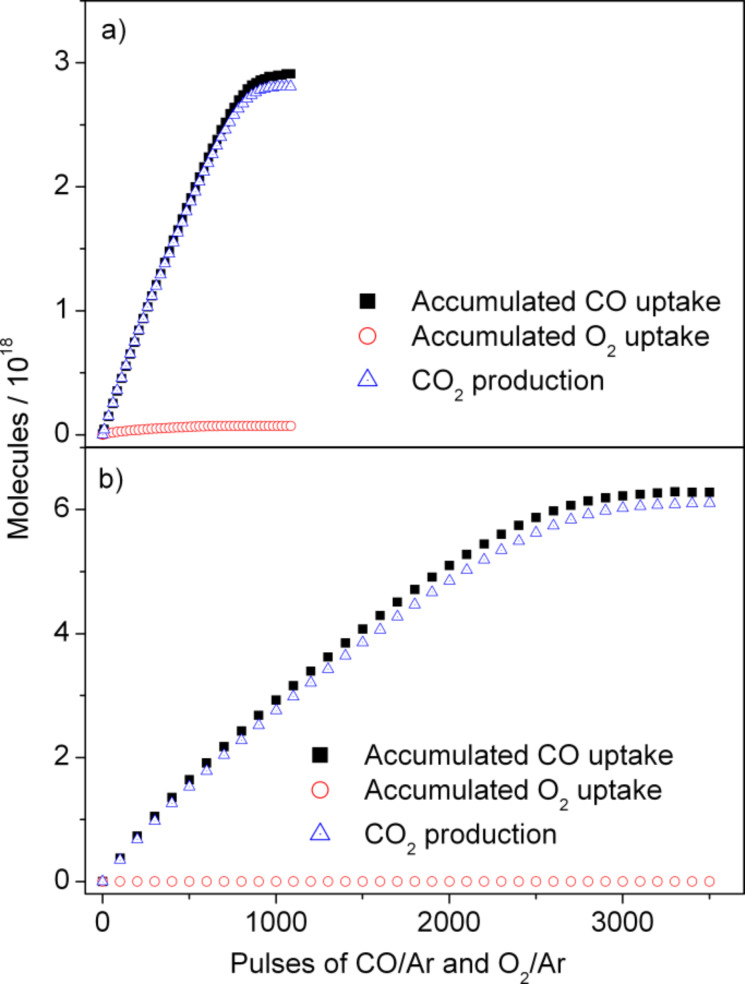
Accumulated uptake of CO and O_2_ as well as CO_2_ formation during simultaneous pulsing of CO/Ar and O_2_/Ar on the fresh (a) NPG(Ag)-4 and (b) NPG(Cu)-2 catalyst in the TAP reactor (2.0 mg NPG catalyst diluted with SiO_2_ (1:10), temperature 30 °C, 1 × 10^16^ molecules per pulse, with CO/Ar and O_2_/Ar = 1:1).

**NPG(Ag):** In these experiments, the sample was exposed to a sequence of simultaneous pulses of CO/Ar and O_2_/Ar at 30 °C reaction temperature. Typical results of these measurements obtained on the NPG(Ag)-4 sample are illustrated in Figure 6a. Apparently, the CO uptake, which is quantitatively identical to the amount of CO_2_ formation, decreased continuously, until after ca. 1100 pulses for 2.0 mg of catalyst it was below the detection level (CO uptake < 1% of the incoming intensity). Meanwhile, no measurable O_2_ uptake could be detected during the whole pulse experiment. Apparently, in this experiment CO oxidation proceeds by a noncatalytic process, through reaction of CO molecules with active oxygen species that were already present on the fresh NPG catalyst. Similar results were obtained also on the other three NPG(Ag) catalysts, with different amounts of CO/O_2_ pulses required to remove the precovered surface oxygen species. In contrast to these findings for the NPG catalysts, steady catalytic activities can be achieved when performing similar single-pulse TAP reactor measurements on supported Au catalysts such as Au/TiO_2_ catalysts [23,46]. The very low catalytic activity of the NPG catalysts in the TAP reactor compared to that of Au/TiO_2_ catalysts under low pressure conditions points to a very low probability for O_2_ activation under these reaction conditions. Most simply, this can be explained by a highly nonlinear pressure dependence for O_2_ activation on the NPG catalysts [12].

**Figure 7:**
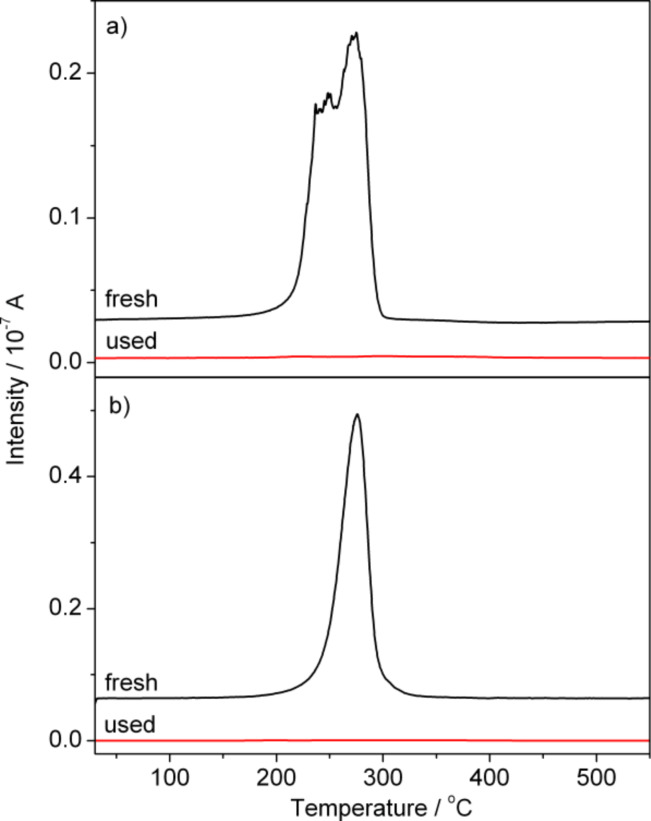
TPD spectra of oxygen species (*m*/*z* = 32) recorded before reaction and after the simultaneous pulsing experiment on the (a) NPG(Ag)-4 and (b) NPG(Cu)-1 catalysts.

In order to determine the nature and the amount of oxygen species present on the NPG(Ag) catalyst surface before and after the pulse reaction, we performed TPD experiments on the fresh NPG(Ag)-4 catalyst as well as after reaction by simultaneous pulses of CO and O_2_ (for details see [12]). Desorption spectra of O_2_ (*m*/*z* = 32) recorded on the fresh NPG(Ag)-4 catalyst showed a pronounced O_2_ desorption peak at around 270 °C (see Figure 7a), which we assigned to the desorption of atomic oxygen species chemisorbed on Au surface sites [12]. In the spectrum recorded after the pulse reaction, however, this peak is essentially absent (see Figure 7a), indicating that the related oxygen species was removed by reaction with CO molecules. The desorption temperature, which is close to that reported for the desorption of atomic oxygen from Au surfaces (270–300 °C [66–69]) and lower than that for the desorption of oxygen from Ag surfaces (322 ± 25 °C [70–72]), points to atomic oxygen species adsorbed on Au sites as the origin for this peak (for the exact nature of the oxygen species see below). This agrees with the conclusions derived from the pulse measurements. Quantitative evaluation of the TPD spectra yields nominal oxygen coverages of 1.5 and 0.04 monolayers (ML) for the fresh and the used NPG(Ag)-4 catalyst, respectively, assuming a specific surface area of 75 m^2^·g^−1^, and a Au surface atom density of 1.4 × 10^15^ atoms·cm^−2^ (surface density on Au(111)) [13]. For the other three NPG(Ag) samples, the results are qualitatively similar, differing only in the amount of oxygen for different fresh samples. In all cases, the active oxygen species already present on the freshly prepared sample surfaces were almost completely depleted after pulse reaction.

**NPG(Cu):** For comparison, we performed similar pulse reaction measurements in the TAP reactor on the NPG(Cu) samples, as representatively illustrated in Figure 6b for NPG(Cu)-2. Again, no measurable catalytic activity was observed, only removal of the precovered surface oxygen species by reaction with CO molecules. The absolute amount of removed surface oxygen, however, is significantly higher than that on the NPG(Ag) samples. For the same amount of NPG catalyst (2 mg), the uptake of CO molecules of the NPG(Cu)-2 sample is about twice as high as that on the NPG(Ag)-4 sample. Considering the comparable surface area, this result is in reasonable agreement with the XPS results, showing an almost doubled fraction of Au^3+^ oxide species on the surface of the NPG(Cu)-2 sample.

Following the pulse reaction, the amount and bond strength of the (remaining) surface oxygen on the NPG(Cu) catalysts was determined in O_2_-TPD experiments. A typical oxygen desorption spectrum on the NPG(Cu)-1 sample is shown in Figure 7b. For comparison, we again include a spectrum of the fresh sample. Similar to our findings for the NPG(Ag) catalysts, essentially all of the surface oxygen was removed on the used sample by reaction with CO. The fresh sample exhibits a single sharp desorption peak with a maximum at 275 °C, equal to that on the NPG(Ag)-4 catalyst (see Figure 7a). Vacuum-annealing-induced reduction of fully oxidized copper films (2 nm thick, grown by physical vapor deposition) has shown that CuO started to form Cu_2_O at around 200 °C and complete reduction to Cu began at 400 °C [73]. On the other hand, temperature-programmed desorption (TPD) experiments performed on Au_2_O_3_ compounds revealed that O_2_ evolves in a temperature range from ca. 250–370 °C, with the highest rate of oxygen evolution at ca. 320 °C, and with no evidence of an intermediate oxidation state of gold, e.g., Au_2_O or AuO [74]. In combination with the rather low content of residual Cu in the NPG(Cu) sample, the peak observed in our TPD measurement (at 275 °C) should be assigned to desorption of atomic oxygen species chemisorbed on Au sites or decomposition of an Au oxide (for details see below).

TPD experiments on the other two NPG(Cu) samples have shown that the desorption temperatures are significantly higher with almost doubled peak intensities (see Supporting Information File 1, Figure S2). Hence, the desorption temperature of the surface oxygen species is strongly affected by the oxygen coverage, i.e., higher coverage leads to higher desorption temperature. This finding is somewhat unexpected, since higher coverages usually destabilize the adsorbed species, resulting in a shift to lower temperatures with increasing coverage. A similar coverage-dependent desorption behavior, however, has also been reported by Gottfried et al. [75] during O_2_-TPD experiments on a Au(110)-(1×2) surface with varying oxygen coverages, i.e., from ca. 0.50 to 1.80 ML. They explained this behavior by an autocatalytic desorption process, based on the assumption that at high oxygen coverages the surface is predominantly covered with O islands possessing a low local desorption rate (tc-phase) [75]. The same may be true in our case; however, an influence of residual Cu on the oxygen desorption from Au cannot be ruled out.

So far we have not specified the nature of the atomic surface oxygen species identified as the active species above. Different types have been proposed. Baker et al. proposed the existence of chemisorbed oxygen in threefold hollow sites, of a 2D surface oxide and of a (subsurface) oxide on Au(111) on the basis of vibrational spectroscopy data and density functional theory based calculations and molecular dynamics simulations [76]. (Interestingly, these authors also remarked that there is no clear definition of the surface oxide and subsurface oxide in the literature.) In a recent high-resolution photoelectron spectroscopy study, Schaefer et al. [77] could indeed identify three different types of atomic oxygen upon interaction of oxygen with nanoporous Au, which they associated with the above species. Considering the onset of oxygen desorption at ca. 200 °C in our TPD measurements, both the chemisorbed oxygen and surface oxide discussed in the paper by Schaefer et al. would be compatible with our findings (little/small loss after 15 min annealing at 150 °C). More information on the nature of this oxygen species, however, cannot be obtained from our results.

#### 2.3 Physical origin of the catalytic activity of NPG catalysts

In the following, we wish to discuss the mechanistic findings derived from the similarities and characteristic differences in the reaction behavior of the NPG(Ag) and NPG(Cu) catalysts. Characteristic features of both NPG(Ag) and NPG(Cu) catalysts are

1. their relatively high activities for reaction under atmospheric pressure conditions in combination with their much lower activity for the activation of O_2_ towards stable adsorbed, atomic oxygen,
2. the pronounced dependence of the reaction rate on the surface content of the less noble metal (Ag, Cu), while the surface area seems to play only a minor role,
3. the distinct initial activation behavior in the initial reaction phase at current reaction conditions, followed by a slow deactivation with time on stream, in combination with an equally pronounced coarsening behavior of the NPG ligament structure and loss of surface area, and variable extents of surface enrichment of the Ag or Cu species during reaction,
4. and finally, the presence of predominantly metallic Ag and Cu species in NPG(Ag) and NPG(Cu) materials under the present reaction conditions.

Consequences arising from these characteristic features shall be discussed in the following.

To (1.): The CO oxidation activity of the NPG catalysts is relatively high, about 3–30% of that of highly active Au/TiO_2_ catalysts when based on the Au mass, and 4–200% when based on the TOF rate (0.28 s^−1^ for 3 wt % Au/TiO_2_ with a mean Au particle size of 3.0 nm). This demonstrates that contributions from a reactive support, e.g., by metal-support interactions or by formation of active sites at the perimeter of the interface between metal and support, are not necessarily required for the reaction. On the other hand, the activation of molecular O_2_ to form stable adsorbed oxygen, which can be regarded as an analogue to the stable surface lattice oxygen proposed as the reactive oxygen species for a number of Au/oxide catalysts under comparable reaction conditions [24–25], is much less efficient on NPG catalysts than on Au/oxide catalysts. In our previous report on the formation of active oxygen on the NPG(Ag)-2 catalyst, we calculated the maximum uptake of oxygen within one pulse to be around 4 × 10^13^ O atoms for 2 mg NPG(Ag), assuming that 10% of the total oxygen deposition during O_2_ pulses occurs in the first pulse [12]. One should note that this maximum uptake of oxygen within one pulse was estimated from a multipulse experiment with 200 O_2_ pulses on a fresh NPG(Ag) sample [12]. For reacted samples (after reaction for 1000 min) or after higher doses of O_2_ (higher amount of O_2_ pulses or O_2_ gas flow at atmospheric pressure) this value is always lower [12–13]. Hence, formation of active oxygen under pulse conditions is at least a factor of 20 less efficient than on supported Au/oxide catalysts under comparable reaction conditions, for which the oxygen uptake within a single O_2_ pulse reaches up to 5 × 10^15^ O atoms (for 10 mg catalyst). Moreover, estimating from these pulse measurements the total amount of reactive atomic oxygen that can be deposited per time unit during the reaction, based on these assumptions, this is at least a factor of 10 lower than the total amount of CO_2_ formed during continuous reaction for NPG(Ag) [12]. The difference is even larger for AuCu-based NPG samples than for the AuAg-based ones. For NPG(Ag), we had previously interpreted this discrepancy as an indication of a pronounced nonlinear pressure effect, leading to a more efficient active oxygen deposition during reaction at atmospheric pressure than during O_2_ pulsing [13]. It is equally possible, however, that different from the reaction mechanism proposed for oxide-supported Au catalysts for reaction above 50 °C, CO oxidation on NPG catalysts does not proceed via a stable adsorbed atomic oxygen species as a reaction intermediate, but by another mechanism. Most likely this would be a mechanism via a CO_ad_···O_2,ad_ intermediate and a CO_ad_ induced dissociation of the adsorbed O_2_, as is likely to occur for oxide-supported catalysts at much lower temperatures [78]. In that case, however, this reaction intermediate was stabilized by the Au-oxide interface (“dual adsorption sites”), which is not present for the NPG catalysts. Therefore, the question of the dominant reaction pathway and the active oxygen species on NPG catalysts is still unresolved. However, the present data provide clear proof that reaction by formation and reactive removal of a stable adsorbed atomic oxygen species is at least a minority pathway.

**Figure 8:**
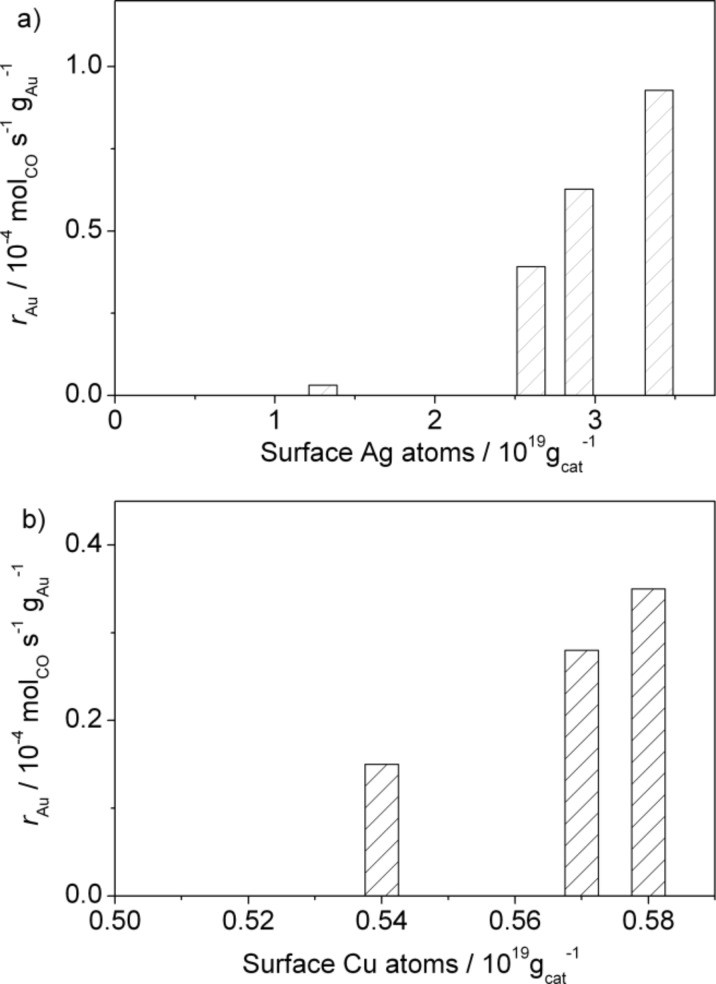
Catalytic activities of various NPG catalysts at 30 °C, plotted against the total amount of (a) surface Ag or (b) surface Cu atoms·g_cat_^−1^ after 1000 min on stream.

To (2.): The above data indicate that the concentration of less noble residues from the original alloy plays an important role in the range of surface contents investigated. To illustrate the influence of surface Ag or Cu atoms on the catalytic activity more quantitatively, we plotted the reaction rates of the different catalysts in the steady state against the corresponding total number of Ag or Cu atoms present on the catalyst surface (after reaction). As shown in Figure 8, there is an almost linear correlation between these two quantities, pointing to the important role of surface Ag or Cu atoms in the catalytic properties of the NPG catalysts. Only the sample NPG(Ag)-1 with the lowest concentration of surface Ag atoms does not fit to this linear trend. Although its activity is rather low compared to that of the other samples, it is still higher than expected from the linear relation of the other three NPG(Ag) samples. Tentatively this may be attributed to an intrinsic activity of Au itself, without major contributions from Ag surface atoms. Obviously, the variation in Cu surface concentrations is too small for an unambiguous identification of a surface concentration effect, but the trend fits well to that observed for NPG(Ag). Interestingly, this also shows that although the activities of the NPG(Cu) catalysts are significantly lower than those of the NPG(Ag) catalysts on average, they are considerably higher than that of the NPG(Ag)-1 sample with the lowest Ag surface content. (Note that the surface Ag content in the latter sample is still almost double that of the surface Cu content of the NPG(Cu) samples.) Surface area effects can be ruled out, since the surface areas under steady-state conditions are comparable for all catalysts.

**Figure 9:**
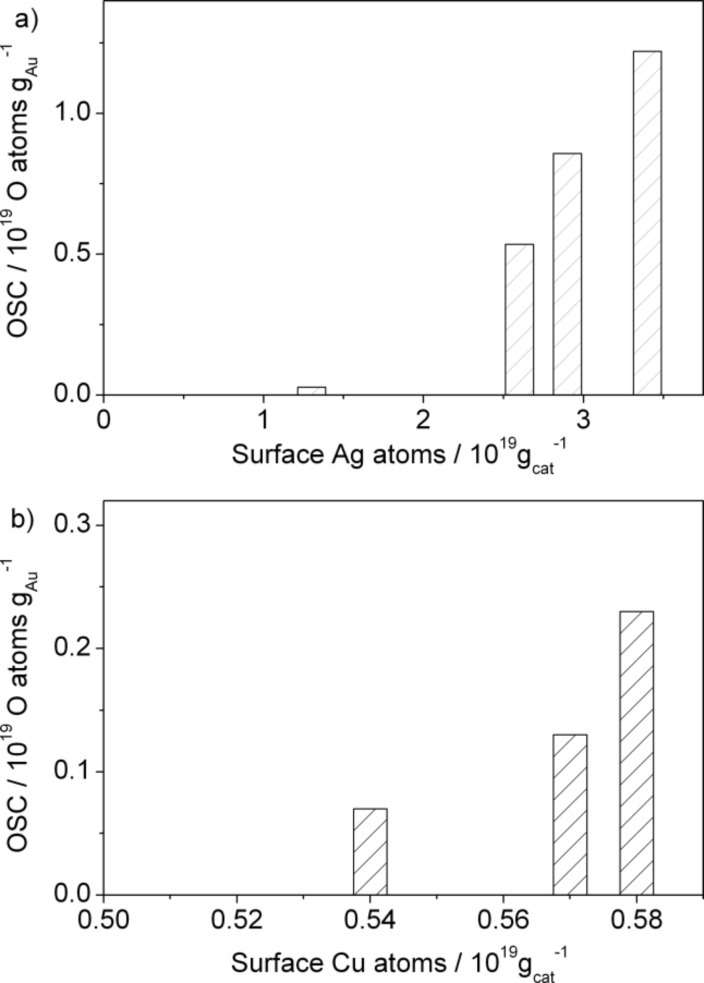
OSC of various NPG catalysts plotted against the total amount of (a) surface Ag or (b) surface Cu atoms·g_cat_^−1^ after 1000 min on stream.

A similar comparison can be performed for the oxygen storage capacity of the various NPG catalysts after reaction for 1000 min. This was measured by exposing the catalysts to a flow of 10% O_2_/N_2_ (20 mL·min^−1^) for 30 min, followed by titration with CO pulses in the TAP reactor. The results are presented in Table 1 and Table 3 and also plotted against the corresponding total number of surface Ag or Cu atoms on the stable catalyst (after reaction) in Figure 9. Again, an almost identical linear correlation was obtained between the OSC and the absolute amount of surface Ag or Cu atoms, and this result strongly suggests that the surface Ag or Cu atoms are directly involved in the activation of molecular oxygen during the oxidation reaction, thus promoting the catalytic activity.

To (3.): Based on the results of the structural characterization described above, we tentatively attribute the initial activity enhancement of the NPG catalysts to the structural rearrangement induced by the removal of the pre-existent oxide species on the fresh samples, where the latter occurs on a much faster time scale, i.e, within a couple of minutes. The pre-existent oxygen species plays an important role in stabilizing the structure of the NPG material [30,40]. It has been suggested that the removal of surface oxygen may lead to two coinciding processes, first the creation of a large transient population of mobile gold adatoms during the reduction process and second, on a longer timescale, the creation of a gold surface that is free of the constraints on diffusivity [40]. In this study, the surface areas of all the Ag- and Cu-containing NPG samples decreased significantly during reaction, by a factor of up to about 7. This yields comparable surface areas after reaction for 1000 min, which, obviously, can neither explain the activation phase in the beginning of the reaction nor the discrepancies in the catalytic activities of various NPG catalysts under steady-state conditions. Hence, the absence of a clear correlation between the activity for CO oxidation and the catalyst surface area indicates that the (total) surface area plays no dominant role in the activity of these samples.

To (4.): Considering the TAP, TPD and XPS results it is likely that under reaction conditions both Ag and Cu are present as metallic species and not as local (surface) oxides, despite the much higher affinity of Cu towards oxygen as compared to Ag. In this case, the NPG catalysts can be considered as bimetallic catalysts, where the presence of the second metal modifies the chemical properties of the main component (Au) and at the same time also introduces its own functionality [79–80]. In fact, the important role of the guest metals Ag or Cu, for example as an active center for O_2_ activation, as a part of active mixed Au*_x_*Me*_y_* ensembles (Me = Ag, Cu), or as a modifier for the Au surface atoms, has been discussed already by both theoretical and experimental research. For instance, Moskaleva et al. [37,81] have investigated the role of Ag in NPG catalysts theoretically by DFT calculations and concluded that Ag plays a decisive role in the activation of oxygen [37]. Experimental studies by several groups have also underlined the role of Ag in AuAg bimetallic systems, including both NPG catalysts [14,30] and supported Au catalysts [35–36]. Significant effects of the Cu content were also reported for AuCu systems [6,38,82]. If the Ag (Cu) atoms (Ag (Cu) ensembles) act as active centers for O_2_ activation, they would take the role of the metal–oxide interface in oxide-supported catalysts [24,83–84]. In that case, the NPG catalysts could be considered as bifunctional catalysts, with the Au atoms supporting CO adsorption/oxidation and Ag (Cu) surface atoms/ensembles supporting O_2_ activation.

## Conclusion

Based on detailed continuous and dynamic reaction measurements of the CO oxidation reaction on unsupported NPG catalysts with systematically varied structural parameters (surface area, ligament size) and surface composition (nature and content of the residual amounts of the less noble metal), we arrived at the following conclusions regarding the mechanism of the CO oxidation reaction on these surfaces and on the physical effects responsible for the activity of these catalysts:

1. The NPG catalysts are highly active for CO oxidation at atmospheric pressure, with Au-mass based activities that are between 3 and 30% of those of highly active Au/TiO_2_ catalysts with 3 nm Au particle size. Hence, the absence of oxide support does not necessarily lead to poor activity for Au catalysts under ambient pressure conditions.
2. In contrast, for reaction in the TAP reactor, the activity decreased to below the detection limit after removal of the precovered surface oxygen.
3. In both cases, the reaction leads to rapid reduction of the pre-existent surface oxygen species present after electrochemical dealloying, until steady-state conditions are reached.
4. The reaction results in a steady coarsening of the NPG structure, evident by a strong increase in the Au ligament size, and enrichment of residual Ag or Cu on the surface, in particular at lower surface contents.
5. For the NPG(Ag) catalysts, the OSC as well as the activity of the NPG catalysts for CO oxidation scale approximately linearly with the number of surface atoms in the stable state after the reaction for 1000 min. Based on these results, we propose that the residual Ag in the NPG catalysts plays an important role in the activation of molecular oxygen and thus the catalytic performance.
6. Correlations between the activity for CO oxidation and the catalyst surface area are weak, considering that the activities of the NPG catalysts are hardly affected by the initial significant loss of surface area, in particular when comparing with the pronounced effects imposed by the surface Ag or Cu content. Obviously, the total surface area plays a less important role for the activity of the NPG samples.
7. Comparison between Ag- and Cu-containing NPG samples suggests that also for NPG(Cu) catalysts the catalytic activity is determined mainly by the amount of the residual less noble metal, but with a higher efficiency for oxygen activation under reaction conditions for Cu compared with Ag.

Moisture in the feed gas has a distinct influence on the catalytic activity of the NPG samples, but neither alters the relative activities of the catalysts, relative to each other, nor the temporal evolution of the activity. The latter is different from observations on supported Au/TiO_2_ catalysts, where the presence of trace amounts of moisture in the feed gas has a clear effect on the deactivation behavior.

## Supporting Information

File 1 Additional XPS and TPD spectra
