# Supplementary material for: Catalytic activity of nanostructured Au: Scale effects versus bimetallic/bifunctional effects in low-temperature CO oxidation on nanoporous Au
Source: Beilstein J Nanotechnol. 2013 Feb 19;4:111–28. doi: 10.3762/bjnano.4.13 (PMC3596058; doi:10.3762/bjnano.4.13)
Supplement: File 1 — Additional XPS and TPD spectra [file Beilstein_J_Nanotechnol-04-111-s001.pdf]

Supporting Information

for

Catalytic activity of nanostructured Au: Scale effects vs bimetallic/bifunctional effects in low temperature CO oxidation on nanoporous Au

Lu-Cun Wang[1], Yi Zhong[2], Haijun Jin[3], Daniel Widmann[1], Jörg Weissmüller[2,4]

and R. Jürgen Behm[1]*

Address: [1]Institute of Surface Chemistry and Catalysis, Ulm University, D-89069 Ulm, Germany, [2]Institut für Werkstoffforschung Helmholtz-Zentrum Geesthacht , D-21502 Geesthacht, Germany, [3]Institute of Metal Research, Chinese Academy of Sciences, 110016, Shenyang, P.R.China and [4]Institut für Werkstoffphysik und Werkstofftechnologie, TU Hamburg-Harburg, D-21073 Hamburg, Germany

Email: Jürgen Behm* - juergen.behm@uni-ulm.de

*Corresponding author

Additional XPS and TPD spectra

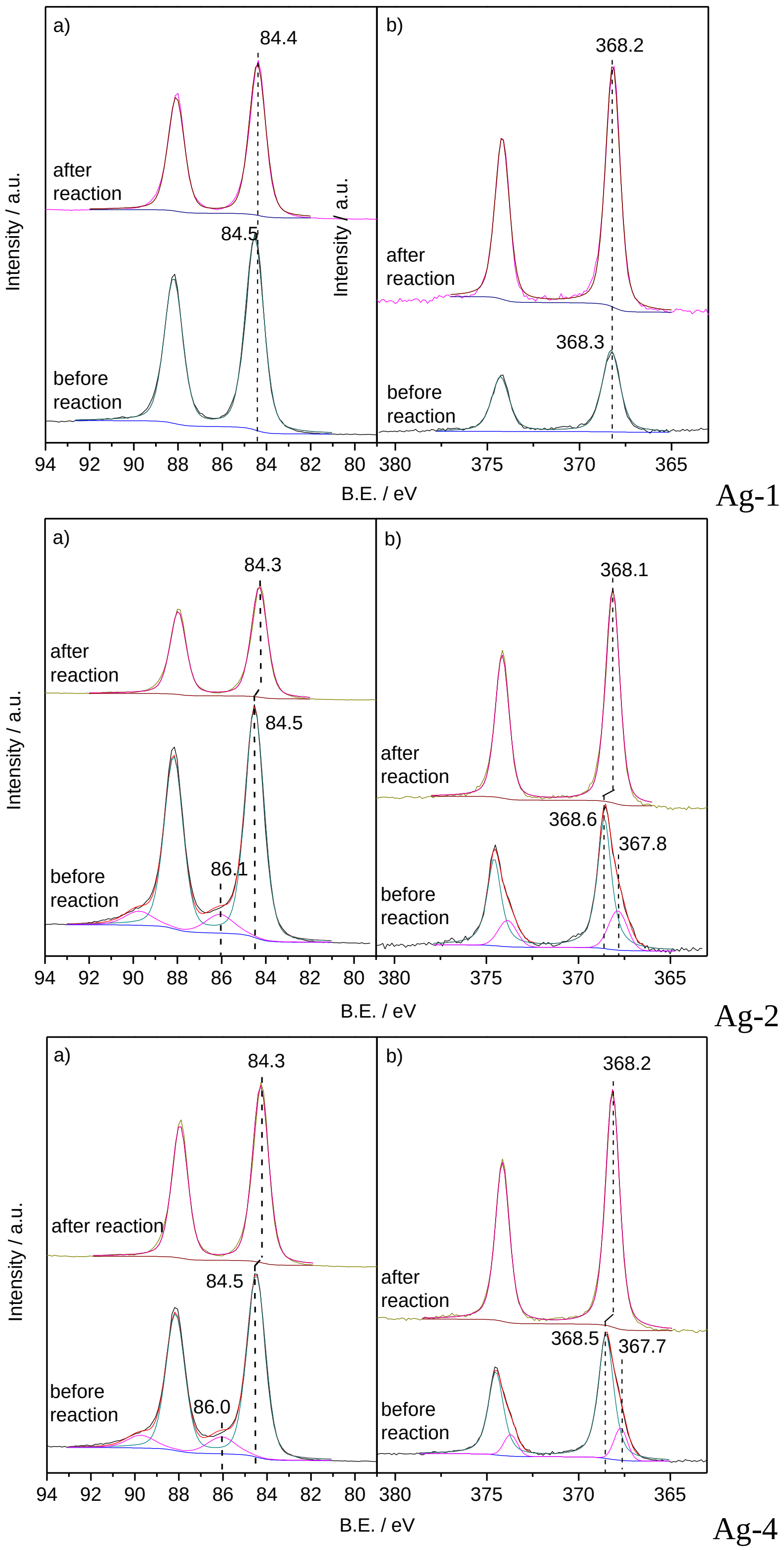

Figure S1: Au(4f) (a) and Ag (3d) (b) XP spectra recorded on various NPG(Ag) catalysts before and after reaction (1000 min on stream).

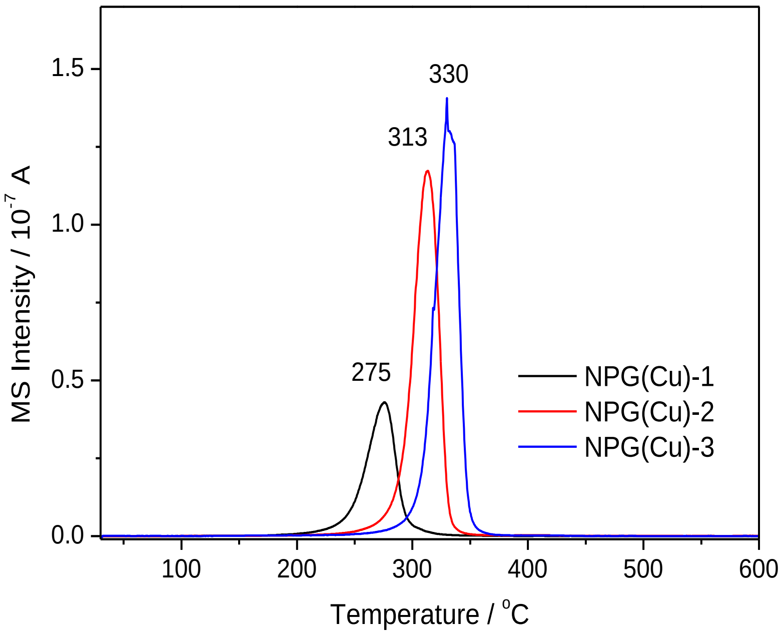

Figure S2: O_2 TPD spectra recorded on various fresh NPG(Cu) catalysts.
